# Assessment of Gastroesophageal Reflux Symptoms and Sleep Quality Among Women in the Nurses’ Health Study II

**DOI:** 10.1001/jamanetworkopen.2023.24240

**Published:** 2023-07-19

**Authors:** Jane Ha, Raaj S. Mehta, Yin Cao, Tianyi Huang, Kyle Staller, Andrew T. Chan

**Affiliations:** 1 Clinical and Translational Epidemiology Unit, Massachusetts General Hospital and Harvard Medical School, Boston; 2 Division of Gastroenterology, Department of Medicine, Massachusetts General Hospital and Harvard Medical School, Boston; 3 Division of Public Health Sciences, Department of Surgery, Washington University School of Medicine in St Louis, Missouri; 4 Division of Gastroenterology, Department of Medicine, Washington University School of Medicine in St Louis, Missouri; 5 Alvin J. Siteman Cancer Center, Washington University School of Medicine in St Louis, Missouri; 6 Division of Sleep Medicine, Harvard Medical School, Boston, Massachusetts; 7 Channing Division of Network Medicine, Brigham and Women’s Hospital and Harvard Medical School, Boston, Massachusetts; 8 Broad Institute of Massachusetts Institute of Technology and Harvard, Cambridge; 9 Department of Immunology and Infectious Diseases, Harvard T.H. Chan School of Public Health, Boston, Massachusetts

## Abstract

**Importance:**

Limited data exist on the association of gastroesophageal reflux (GER) symptoms with sleep quality.

**Objective:**

To prospectively investigate the association between GER symptoms and sleep quality.

**Design, Setting, and Participants:**

This prospective cohort study included data from the Nurses’ Health Study II of female nurses in the US. Participants self-reported the frequency and duration of GER symptoms beginning June 2005, with updates every 4 years through June 2015. Follow-up was completed June 2019, and data were analyzed from November 15, 2022, to June 4, 2023.

**Exposures:**

Frequency and duration of GER symptoms.

**Main Outcomes and Measures:**

Poor sleep quality was assessed in 2017 through a modified Pittsburgh Sleep Quality Index, which included difficulty in falling asleep, restlessness of sleep, daytime sleepiness, sleep disturbance, and sleep duration. Relative risk (RR) for poor sleep quality and individual components of poor sleep quality was estimated according to the frequency and duration of GER symptoms.

**Results:**

Among 48 536 women (median age, 59 years [range, 48-69 years]), 7929 (16.3%) developed poor sleep quality during 4 years of follow-up. Compared with those with GER symptoms less than once a month, the multivariable RR for poor sleep quality among women with GER symptoms more than once a week was 1.53 (95% CI, 1.45-1.62). Women who had GER symptoms once or more a week for more than 7 years had an RR of 1.36 (95% CI, 1.30-1.43) compared with women who had not had GER symptoms once or more a week. The frequency and duration of GER symptoms were significantly associated with each individual component of poor sleep quality; for example, the multivariable RRs for GER symptoms 2 or more times per week compared with no GER symptoms were 1.49 (95% CI, 1.39-1.58) for difficulty in falling asleep, 1.47 (95% CI, 1.39-1.56) for excessive daytime sleepiness, and 1.44 (95% CI, 1.36-1.53) for restlessness of sleep.

**Conclusions and Relevance:**

In this prospective cohort study of female nurses in the Nurses’ Health Study II, the frequency and duration of GER symptoms were associated with subsequent risk of poor sleep quality. The findings suggest that effective treatment of GER disease may be important not only for improvement of symptoms but also for the reduction of comorbidities associated with poor sleep quality.

## Key Points

**Question:**

Are gastroesophageal reflux symptoms associated with subsequent risk of poor sleep quality among women?

**Findings:**

In this cohort study of 48 536 women, gastroesophageal reflux symptoms, particularly those that were more frequent and of longer duration, were associated with an increased risk of developing poor sleep quality among women.

**Meaning:**

The findings suggest that effective treatment of gastroesophageal reflux disease may be important not only for improvement of symptoms but also for the reduction of comorbidities associated with poor sleep quality.

## Teaser

This cohort study assesses the association between gastroesophageal reflux symptoms and risk of subsequent poor sleep quality among women in the Nurses’ Health Study II.

## Introduction

Approximately 20% of the US population experiences gastroesophageal reflux (GER) symptoms at least once a week, and the worldwide prevalence of GER disease (GERD) has been increasing.^1^ Beyond its association with quality of life, GERD is also associated with long-term complications, including Barrett esophagus and esophageal adenocarcinoma.^2^

Sleep plays a critical role in metabolism and energy homeostasis. Impaired sleep affects functional performance and overall quality of life.^3^ Moreover, both sleep duration and quality are associated with various health outcomes, including type 2 diabetes,^4,5^ cardiovascular diseases,^6,7^ and even higher mortality.^8,9^ Although sleep hygiene and comorbid conditions are known to be associated with sleep quality, data on additional risk factors are limited.

Growing evidence supports an association between GER symptoms and poor sleep quality. However, several previous studies are limited by a cross-sectional design and a lack of data on frequency and duration of symptoms.^10,11,12^ Thus, we prospectively evaluated the association of the frequency and duration of GER symptoms with subsequent risk of poor sleep quality. We also assessed whether regular use of a proton pump inhibitor (PPI) and/or histamine-2 receptor antagonists (H2RAs) modified the association between GER symptoms and sleep quality.

## Methods

### Study Population

In this cohort study, we examined data collected among women enrolled in the Nurses’ Health Study II, an ongoing, prospective study that originally enrolled 116 429 female participants who have been followed up since 1989, with over 90% follow-up. Information on medical history, diagnosis of new diseases, and lifestyle behavior has been collected through mailed questionnaires administered biennially. The study protocol was approved by the institutional review board of the Brigham and Women’s Hospital and the Harvard T.H. Chan School of Public Health, which deemed the participants’ completion of questionnaires to be considered as implied consent. The study was conducted in accordance with the Strengthening the Reporting of Observational Studies in Epidemiology (STROBE) reporting guideline.

### Assessment of GER Symptoms

Beginning June 2005, participants reported whether they experienced acid reflux or heartburn symptoms and the frequency of the symptoms every 4 years through June 2015. Follow-up was completed in June 2019. Response categories included “none in the past year,” “less than 1/month,” “about 1/month,” “about once/week,” “several times/week,” or “daily.” Duration of having GER symptoms once or more a week^13^ was calculated based on updated responses to the GER question on follow-up questionnaires.

### Assessment of Sleep Quality

From June 2017 to June 2019, participants responded to a detailed questionnaire adapted from the Pittsburgh Sleep Quality Index, the most rigorously validated tool for evaluation of overall sleep quality,^14^ that included 5 components: difficulty in falling asleep, restlessness of sleep, daytime sleepiness, sleep disturbance, and sleep duration, each scoring 0 (no difficulty) to 3 (severe difficulty). Participants with an overall score of more than 7 were considered to have poor sleep quality, and each component was also individually assessed as a binary outcome (eMethods in Supplement 1).

### Assessment of Covariates

We additionally collected data on known and putative risk factors for poor sleep quality, including age; race (American Indian, Asian, Black, Native Hawaiian, White, multiracial, and not reported; ascertained by self-report); body mass index (BMI; calculated as weight in kilograms divided by height in meters squared); menopausal hormone use; smoking status; alcohol consumption; presence of cancer, congestive heart failure, diabetes, asthma, hyperthyroidism, hypothyroidism, depression, self-reported depression and anxiety symptoms based on the Patient Health Questionnaire-4, urinary incontinence, and hot flushing; intake of caffeinated beverage and decaffeinated beverage; Alternative Healthy Eating Index score; physical activity in metabolic equivalent tasks per week; PPI and/or H2RA use; diuretics use; and the number of months working a night shift in the past 2 years. Details are described in the eMethods in Supplement 1.

### Statistical Analysis

Data were analyzed from November 15, 2022, to June 4, 2023. Differences in overall and individual components of sleep quality scores across the different frequency and duration groups for GER symptoms were tested using analysis of covariance adjusting for covariates. For missing covariates, we carried forward nonmissing values from previous questionnaires and then assigned median values for continuous variables. The missingness was less than 5% for all variables. Log-binomial regression models were used to estimate relative risks (RRs) and 95% CIs for poor sleep quality and individual components according to the frequency of GER symptoms (<1 time per month, 1-3 times per month, 1 time per week, ≥2 times per week) and the duration of frequent (defined as ≥1 time per week) GER symptoms (no frequent symptoms, <4 years, 4-7 years, or ≥8 years).^15^ To test for trend, we used continuous values for the number of mean days with GER symptoms per month and the number of years with GER symptoms reported by participants.

We evaluated whether regular use of PPIs and/or H2RAs modified the association of the frequency of GER symptoms with overall poor sleep quality and the individual components. Additional stratified analyses for the association of the frequency and duration of GER symptoms with overall poor sleep quality were performed by age (<60 or ≥60 years), BMI (<25 or ≥25), smoking status (never, past, or current), and physical activity (<20 or ≥20 metabolic equivalent tasks per week). Interactions were tested by the likelihood ratio test of the cross-product term. Sensitivity analyses excluding women who worked a night shift for 1 or more months over the past 2 years were performed to account for the direction of the association potentially being reversed.

All analyses were adjusted for the a priori covariates. The significance threshold was *P* < .05 using 2-sided tests for the analyses. SAS, version 9.4 (SAS Institute Inc) was used for statistical analyses.

## Results

Among the 79 124 women who returned data on GER symptoms on the 2013 questionnaire, we excluded those who reported sleep apnea or sleep problems (self-reported trouble falling asleep, sleep disturbance, daytime sleepiness, or restlessness of sleep) in 2013 (n = 19 598) and those who did not return the 2017 questionnaire (n = 10 100) or provide detailed information on sleep quality in 2017 (n = 890), leaving 48 536 women for analysis (eFigure in Supplement 1). Among the 48 536 women aged 48 to 69 years (median age, 59 years), 0.1%% were American Indian; 1.2%, Asian; 1.0%, Black; 0.1% Native Hawaiian; 95.2%, White; and 1.7% multiracial. Race was not reported for 0.7% of women. A total of 7726 women (15.9%) had GER symptoms at least twice per week at baseline assessment, and 7929 (16.3%) developed poor sleep quality during 4 years of follow-up. Age-standardized characteristics of the study population according to the frequency of GER symptoms are shown in Table 1. Women with more frequent GER symptoms were more likely to have a higher BMI, be less physically active, and have asthma and depression. The proportion of participants reporting regular use of PPIs and/or H2RAs was 48.2% among those who reported GER symptoms more than once a week compared with only 6.4% among people with GER symptoms less than once a month. The mean (SD) score of overall sleep, with a higher score indicating poorer sleep quality, was 4.7 (2.5) among women who had GER symptoms less than once per month and 5.6 (2.7) among those with symptoms 2 or more times per week. Increasing frequency of GER symptoms was associated with a progressive decline in sleep quality as measured by sleep score (eTable 1 in Supplement 1).

**Table 1.** Characteristics of Participants According to Frequency of GER Symptoms in the Nurses’ Health Study II in 2013

Characteristic	Participants by frequency of GER symptoms^a^			
	<1 Time/mo (n = 28 991)	1-3 Times/mo (n = 3436)	1 Time/wk (n = 8383)	≥2 Times/wk (n = 7726)
Age, mean (SD), y	58.7 (4.6)	58.6 (4.5)	58.8 (4.7)	58.9 (4.7)
Body mass index, median (IQR)^b^	24.8 (22.2-28.5)	26 (23.1-30.1)	26.9 (23.7-31.1)	27.3 (24-31.4)
Race				
American Indian	13 (<0.1)	0	5 (0.1)	8 (0.1)
Asian	424 (1.5)	31 (0.9)	90 (1.1)	51 (0.7)
Black	321 (1.1)	33 (1.0)	74 (0.9)	69 (0.9)
Native Hawaiian	27 (0.1)	2 (0.1)	5 (0.1)	3 (<0.1)
White	27 510 (94.9)	3293 (95.8)	7995 (95.4)	7404 (95.8)
Multiracial	477 (1.6)	51 (1.5)	152 (1.8)	136 (1.8)
Not reported	219 (0.8)	26 (0.7)	61 (0.7)	56 (0.7)
Smoking status				
Never	19 762 (68.2)	2215 (64.5)	5333 (63.6)	4930 (63.8)
Past	8156 (28.1)	1079 (31.4)	2660 (31.7)	2446 (31.7)
Current	1073 (3.7)	142 (4.1)	390 (4.6)	350 (4.5)
Smoking, median (IQR), pack-years	0 (0-4)	0 (0-5)	0 (0-6)	0 (0-6)
Alcohol use, median (IQR), g/d	2.6 (0.4-9)	2.8 (0.6-9.4)	2.5 (0.4-8.4)	2.5 (0.4-7.8)
Caffeinated beverage, median (IQR), servings/d	2.2 (1.1-3)	2.2 (1.2-3.1)	2.2 (1.2-3.2)	2.2 (1.2-3.2)
Decaffeinated beverage, median (IQR), servings/d	0.4 (0.1-1)	0.4 (0.1-1.1)	0.4 (0.1-1.1)	0.4 (0.1-1.1)
Alternative Healthy Eating Index score, median (IQR)^c^	56.7 (50.5-64.9)	56.3 (49.7-63.2)	56.2 (48.6-62.3)	56.3 (48.6-62.3)
Physical activity, median (IQR), MET/wk	22 (11.2-38.4)	18.4 (8.9-32.8)	17.1 (7.9-31)	16.3 (7.8-30.7)
MHT use				
Premenopausal	4152 (14.3)	503 (14.6)	1161 (13.8)	1057 (13.7)
Never	12 958 (44.7)	1454 (42.3)	3361 (40.1)	2974 (38.5)
Current	4432 (15.3)	602 (17.5)	1447 (17.3)	1334 (17.3)
Past	7449 (25.7)	877 (25.5)	2414 (28.8)	2361 (30.6)
Regular PPI and/or H2RA use	1851 (6.4)	630 (18.3)	2684 (32.0)	3723 (48.2)
Diuretics use	3040 (10.5)	440 (12.8)	1168 (13.9)	1145 (14.8)
Night shifts for ≥1 mo in past 2 y	1368 (4.7)	187 (5.5)	477 (5.7)	414 (5.4)
Comorbidities				
Any cancer	1954 (6.7)	241 (7.0)	600 (7.2)	557 (7.2)
Congestive heart failure	86 (0.3)	15 (0.4)	43 (0.5)	55 (0.7)
Diabetes	1296 (4.5)	220 (6.4)	572 (6.8)	569 (7.4)
Asthma	2170 (7.5)	342 (10.0)	1034 (12.3)	1054 (13.6)
Hyperthyroidism	533 (1.8)	57 (1.7)	191 (2.3)	163 (2.1)
Hypothyroidism	4773 (16.5)	584 (17.0)	1522 (18.2)	1447 (18.7)
Depression	3091 (10.7)	479 (13.9)	1301 (15.5)	1379 (17.9)
Urinary incontinence	16 865 (58.2)	2224 (64.7)	5254 (62.7)	4813 (62.3)
Hot flushing symptoms	12 027 (41.5)	1571 (45.7)	4049 (48.3)	3628 (47.0)
Self-reported psychiatric factors^d^				
Sadness	2767 (9.5)	371 (10.8)	1036 (12.4)	1097 (14.2)
Discouragement	3237 (11.2)	484 (14.1)	1193 (14.2)	1284 (16.6)
Lack of interest in work, hobbies, or relationship	2483 (8.6)	348 (10.1)	934 (11.1)	1004 (13.0)
Anxiety	2048 (7.1)	332 (9.7)	780 (9.3)	816 (10.6)

Abbreviations: GER, gastroesophageal reflux; H2RA, histamine-2 receptor antagonist; MET, metabolic equivalent tasks; MHT, menopausal hormone therapy; PPI, proton pump inhibitor.

^a^ Data are reported as number (percentage) of participants unless otherwise indicated.

^b^ Calculated as weight in kilograms divided by height in meters squared.

^c^ Scores range from 0 to 110, with higher scores indicating a healthier diet.

^d^ Based on the Patient Health Questionnaire-4.

Compared with women who reported GER symptoms less than once a month, the multivariable-adjusted RRs for poor sleep quality, defined as an overall sleep score higher than 7, were 1.15 (95% CI, 1.06-1.25) for those with symptoms 1 to 3 times per month, 1.31 (95% CI, 1.24-1.38) for symptoms once per week, and 1.53 (95% CI, 1.45-1.62) for symptoms 2 or more times per week (*P* < .001 for trend) (Table 2). For each of the individual components of sleep quality, the multivariable RRs for GER symptoms 2 or more times per week compared with no GER symptoms were 1.49 (95% CI, 1.39-1.58) for difficulty in falling asleep, 1.47 (95% CI, 1.39-1.56) for excessive daytime sleepiness, and 1.44 (95% CI, 1.36-1.53) for restlessness of sleep. Having more frequent GER symptoms was significantly associated with higher risk of poor sleep quality across all individual components among both women who regularly used or did not use PPIs and/or H2RAs (Figure). However, risk of poor sleep quality was greater among women who were not using regular PPIs and/or H2RAs (multivariable RR for GER symptoms 2 or more times per week compared with no GER symptoms: 1.61; 95% CI, 1.51-1.71) than among women who were using regular PPIs and/or H2RA (multivariable RR, 1.31; 95% CI, 1.16-1.47) (*P* < .001 for interaction).

**Table 2.** Association Between the Frequency of GER Symptoms and Sleep Quality

Sleep quality	Frequency of GER symptoms				*P* value for trend^a^
	<1 Time/mo (n = 28 991)	1-3 Times/mo (n = 3436)	1 Time/wk (n = 8383)	≥2 Times/wk (n = 7726)	
**Poor sleep quality** ^b^					
Events, No.	3944	574	1621	1790	NA
Age-adjusted RR (95% CI)	1 [Reference]	1.23 (1.13-1.33)	1.43 (1.36-1.51)	1.72 (1.64-1.81)	<.001
Multivariable-adjusted RR (95% CI)^c^	1 [Reference]	1.15 (1.06-1.25)	1.31 (1.24-1.38)	1.53 (1.45-1.62)	<.001
**Difficulty in falling asleep**					
Events, No.	3152	439	1296	1422	NA
Age-adjusted RR (95% CI)	1 [Reference]	1.17 (1.07-1.29)	1.42 (1.34-1.51)	1.70 (1.60-1.80)	<.001
Multivariable-adjusted RR (95% CI)^c^	1 [Reference]	1.10 (1.00-1.21)	1.28 (1.21-1.36)	1.49 (1.39-1.58)	<.001
**Excessive daytime sleepiness**					
Events, No.	3382	499	1400	1508	NA
Age-adjusted RR (95% CI)	1 [Reference]	1.24 (1.14-1.35)	1.44 (1.36-1.53)	1.69 (1.60-1.79)	<.001
Multivariable-adjusted RR (95% CI)^c^	1 [Reference]	1.15 (1.05-1.25)	1.31 (1.24-1.39)	1.47 (1.39-1.56)	<.001
**Restlessness of sleep**					
Events, No.	3866	534	1490	1608	NA
Age-adjusted RR (95% CI)	1 [Reference]	1.16 (1.07-1.26)	1.34 (1.27-1.42)	1.58 (1.50-1.66)	<.001
Multivariable-adjusted RR (95% CI)^c^	1 [Reference]	1.10 (1.01-1.20)	1.24 (1.17-1.31)	1.44 (1.36-1.53)	<.001
**Sleep disturbance**					
Events, No.	6773	897	2394	2476	NA
Age-adjusted RR (95% CI)	1 [Reference]	1.12 (1.05-1.18)	1.23 (1.18-1.28)	1.38 (1.33-1.44)	<.001
Multivariable-adjusted RR (95% CI)^c^	1 [Reference]	1.08 (1.02-1.15)	1.19 (1.14-1.24)	1.33 (1.27-1.39)	<.001
**Short sleep duration** ^d^					
Events, No.	7245	879	2296	2284	NA
Age-adjusted RR (95% CI)	1 [Reference]	1.02 (0.96-1.09)	1.11 (1.06-1.15)	1.20 (1.15-1.25)	<.001
Multivariable-adjusted RR (95% CI)^c^	1 [Reference]	1.01 (0.95-1.07)	1.08 (1.04-1.12)	1.17 (1.12-1.22)	<.001

Abbreviations: GER, gastroesophageal reflux; MHT, menopausal hormone therapy; NA, not applicable; RR, relative risk.

^a^ To test *P* values for trend, we assigned continuous values for the number of mean days with GER symptoms per month.

^b^ Defined as overall sleep score greater than 7.

^c^ Multivariable model was adjusted for age (continuous); body mass index (continuous); menopausal status or menopausal hormone use (premenopausal, MHT never user, MHT current user, or MHT past user); smoking status (never, past, or current); race (American Indian, Asian, Black, Native Hawaiian, White, multiracial, and not reported); presence of cancer (yes, no), congestive heart failure (yes, no), diabetes (yes, no), asthma (yes, no), hyperthyroidism (yes, no), hypothyroidism (yes, no), depression (yes, no), self-reported depression and anxiety symptoms (ordinal), urinary incontinence (yes, no), and hot flushing (yes, no); alcohol consumption (continuous); intake of caffeinated beverage (continuous) and decaffeinated beverage (continuous); physical activity (continuous); diuretics use (yes, no); proton pump inhibitor and/or histamine-2 receptor antagonist use (yes, no); number of months working night shifts in past 2 years (≥1 month, <1 month); and Alternative Healthy Eating Index score (continuous).

^d^ Sleep for less than 7 hours.

**Figure.**
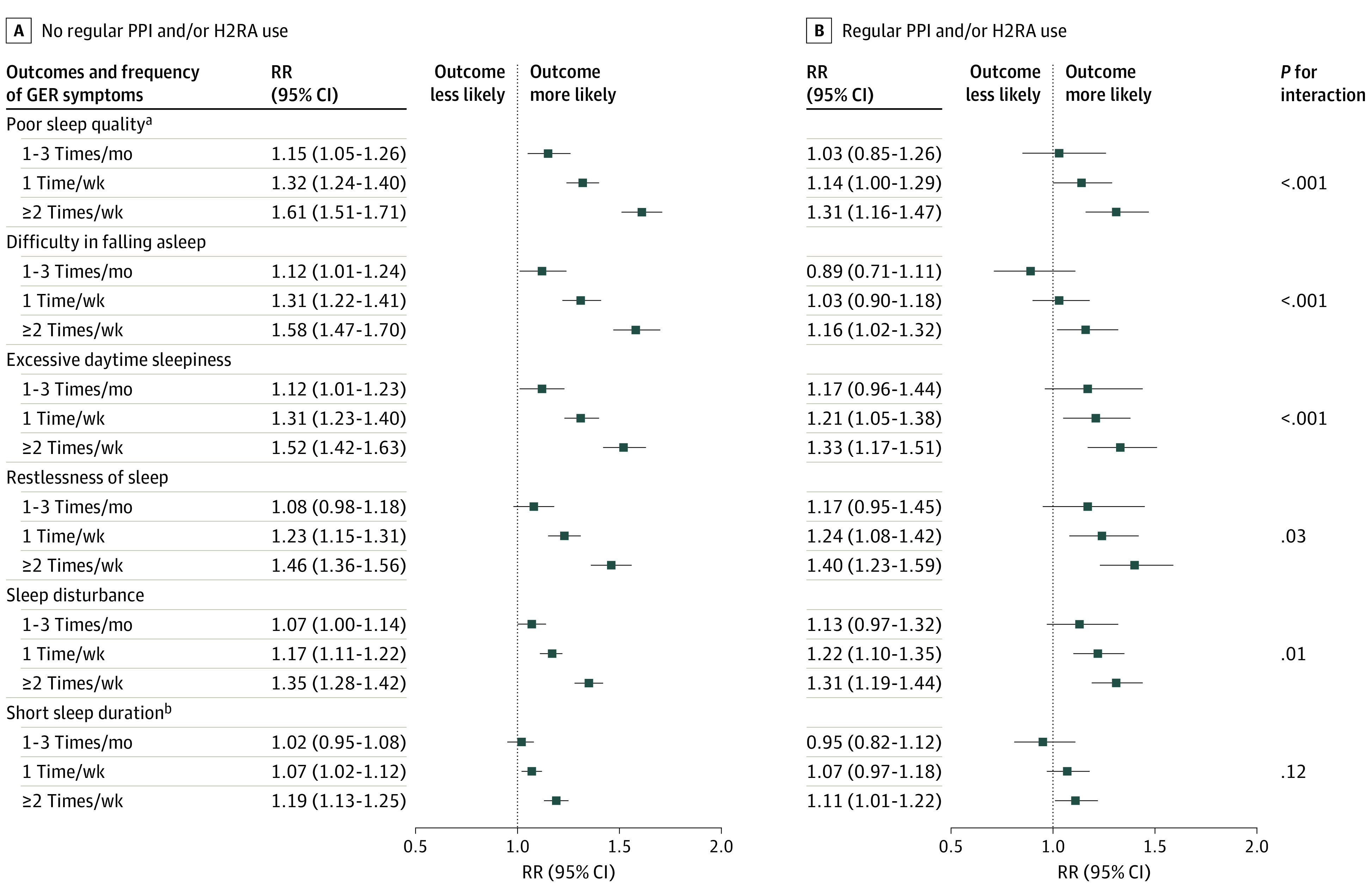
Association Between the Frequency of Gastroesophageal Reflux (GER) Symptoms and Sleep Quality With or Without Regular Proton Pump Inhibitor (PPI) and/or Histamine-2 Receptor Antagonist (H2RA) Use Multivariable models were adjusted for age (continuous); body mass index (continuous); menopausal status or menopausal hormone use (premenopausal, menopausal hormone therapy [MHT] never user, MHT current user, or MHT past user); smoking status (never, past, or current); race (American Indian, Asian, Black, Native Hawaiian, White, multiracial, and not reported); presence of cancer (yes, no), congestive heart failure (yes, no), diabetes (yes, no), asthma (yes, no), hyperthyroidism (yes, no), hypothyroidism (yes, no), depression (yes, no), self-reported depression and anxiety symptoms (ordinal), urinary incontinence (yes, no), and hot flushing (yes, no); alcohol consumption (continuous); intake of caffeinated beverage (continuous) and decaffeinated beverage (continuous); Alternate Healthy Eating Index score (continuous); physical activity (continuous); diuretics use (yes, no); and number of months working night shifts in past 2 years (≥1 month, <1 month). Markers indicate estimates, with horizontal lines indicating 95% CIs. RR indicates relative risk. ^a^Defined as overall sleep score greater than 7. ^b^Sleep for less than 7 hours.

We also evaluated whether the duration of having GER symptoms once or more per week was associated with subsequent risk of poor sleep quality. Women who had GER symptoms once or more per week for more than 8 years had a mean (SD) overall sleep score of 5.4 (2.6), while those who had not had GER symptoms once or more per week had a mean (SD) score of 4.7 (2.5) (eTable 2 in Supplement 1). Compared with those who had not had GER symptoms once or more per week, the RRs for poor sleep quality were 1.22 (95% CI, 1.12-1.33) for women with GER symptoms once or more per week for less than 4 years, 1.38 (95% CI, 1.28-1.49) for those with symptoms for 4 to 7 years, and 1.36 (95% CI, 1.30-1.43) for those with symptoms for 8 or more years (Table 3). The duration of frequent GER symptoms was associated with each of the individual components of poor sleep quality.

**Table 3.** Association Between the Duration of GER Symptoms Once or More Per Week and Sleep Quality

Sleep quality	Duration of GER symptoms once or more a wk				*P* value for trend^a^
	None (n = 28 283)	<4 y (n = 2699)	4-7 y (n = 3325)	≥8 y (n = 14 229)	
**Poor sleep quality** ^b^					
Events, No.	3839	484	683	2923	NA
Age-adjusted RR (95% CI)	1 [Reference]	1.33 (1.22-1.45)	1.52 (1.41-1.64)	1.53 (1.46-1.60)	<.001
Multivariable-adjusted RR (95% CI)^c^	1 [Reference]	1.22 (1.12-1.33)	1.38 (1.28-1.49)	1.36 (1.30-1.43)	<.001
**Difficulty in falling asleep**					
Events, No.	3036	396	537	2340	NA
Age-adjusted RR (95% CI)	1 [Reference]	1.37 (1.24-1.51)	1.51 (1.38-1.64)	1.54 (1.46-1.61)	<.001
Multivariable-adjusted RR (95% CI)^c^	1 [Reference]	1.24 (1.12-1.37)	1.35 (1.24-1.47)	1.35 (1.28-1.42)	<.001
**Excessive daytime sleepiness**					
Events, No.	3278	429	584	2498	NA
Age-adjusted RR (95% CI)	1 [Reference]	1.38 (1.26-1.51)	1.53 (1.41-1.65)	1.54 (1.46-1.61)	<.001
Multivariable-adjusted RR (95% CI)^c^	1 [Reference]	1.26 (1.15-1.38)	1.38 (1.28-1.50)	1.34 (1.27-1.41)	<.001
**Restlessness of sleep**					
Events, No.	3790	481	636	2591	NA
Age-adjusted RR (95% CI)	1 [Reference]	1.34 (1.23-1.46)	1.44 (1.33-1.55)	1.38 (1.32-1.44)	<.001
Multivariable-adjusted RR (95% CI)^c^	1 [Reference]	1.25 (1.14-1.36)	1.32 (1.22-1.42)	1.25 (1.19-1.31)	<.001
**Sleep disturbance**					
Events, No.	6646	751	1008	4135	NA
Age-adjusted RR (95% CI)	1 [Reference]	1.19 (1.11-1.27)	1.30 (1.23-1.37)	1.25 (1.21-1.29)	<.001
Multivariable-adjusted RR (95% CI)^c^	1 [Reference]	1.14 (1.07-1.22)	1.25 (1.18-1.32)	1.20 (1.16-1.24)	<.001
**Short sleep duration** ^d^					
Events, No.	7088	687	948	3981	NA
Age-adjusted RR (95% CI)	1 [Reference]	1.03 (0.86-1.24)	1.15 (1.08-1.21)	1.13 (1.10-1.17)	<.001
Multivariable-adjusted RR (95% CI)^c^	1 [Reference]	0.99 (0.93-1.06)	1.12 (1.06-1.18)	1.10 (1.07-1.15)	<.001

Abbreviations: GER, gastroesophageal reflux; MHT, menopausal hormone therapy; NA, not applicable; RR, relative risk.

^a^ To test *P* values for trend, we assigned continuous values for the number of years with GER symptoms once or more per week.

^b^ Defined as overall sleep score greater than 7.

^c^ The multivariable model was adjusted for age (continuous); body mass index (continuous); menopausal status or menopausal hormone use (premenopausal, MHT never user, MHT current user, or MHT past user); smoking status (never, past, or current); race (American Indian, Asian, Black, Native Hawaiian, White, multiracial, and not reported); presence of cancer (yes, no), congestive heart failure (yes, no), diabetes (yes, no), asthma (yes, no), hyperthyroidism (yes, no), hypothyroidism (yes, no), depression (yes, no), self-reported depression and anxiety symptoms (ordinal), urinary incontinence (yes, no), and hot flushing (yes, no); alcohol consumption (continuous); intake of caffeinated beverage (continuous) and decaffeinated beverage (continuous); physical activity (continuous); diuretics use (yes, no); proton pump inhibitor and/or histamine-2 receptor antagonist use (yes, no); number of months working night shifts in past 2 years (≥1 month, <1 month); and the Alternative Healthy Eating Index score (continuous).

^d^ Sleep for less than 7 hours.

We examined the association between duration and frequency of GER symptoms and poor sleep quality across subgroups defined according to age, BMI, smoking status, or physical activity (eTables 3 and 4 in Supplement 1). There were no statistically significant differences in risk within subgroups with the exception of higher risk of poor sleep quality among older women (age with ≥60 years) with a duration of GER symptoms once or more per week. Sensitivity analyses excluding night shift workers showed consistent associations between the frequency (RR, 1.52 [95% CI, 1.44-1.61] among women with GER symptoms ≥2 times per week compared with symptoms <1 time per month) and duration (RR, 1.37 [95% CI, 1.30-1.44] among women with frequent GER symptoms for ≥8 years compared with those without frequent GER symptoms) of GER symptoms and poor sleep quality.

## Discussion

In this prospective cohort study, we found that GER symptoms were associated with an increase in subsequent risk of poor sleep quality. Although risk was somewhat attenuated among women who regularly used PPIs and/or H2RAs, the risk of poor sleep quality remained significantly higher among those who experienced GER symptoms at least once a week.

Our data support a potential association between GER symptoms and the subsequent development of poor sleep quality. Previous studies have reported a higher prevalence of poor sleep quality in patients with GERD. A cross-sectional survey showed that 68.3% of patients with GERD reported sleep difficulties, with nighttime GER symptoms associated with difficulty in falling asleep and sleep disturbance.^10^ Another population-based case-control study showed significant associations between the risk of GERD and the presence of insomnia, sleeplessness, and difficulty in falling asleep.^11^ A study in Sweden using surveys conducted 10 years apart reported that a higher risk of daytime sleepiness was associated with persistent nocturnal GER symptoms among women (odds ratio, 3.0; 95% CI, 1.5-5.9).^16^

Several mechanisms may underlie the association between GER symptoms and sleep quality. Perhaps the most plausible explanation is that nocturnal GER symptoms may trigger sleep disturbances. However, data have been inconsistent in showing whether physiologically measured reflux events precede nocturnal arousals or awakenings.^17,18,19^ Moreover, some studies have suggested a potential bidirectional association between GERD and sleep quality.^20,21^ There has been literature supporting more frequent reflux symptoms during sleep, which are thought to be associated with recumbent posture and physiologic change of esophageal motility. During sleep, the acid contents remain longer due to fewer effects of gravity against the reflux, the reduction in saliva production, swallowing, and peristalsis of the esophagus that help to clear refluxed contents.^22,23^ Improvement in subjective and objective reflux symptoms with bed head elevation further supports the influence of recumbent position.^24,25^ Moreover, sleep stages have been shown to be associated with reflux, with more events occurring during a daytime nap than during nocturnal sleep^26^ and in sleep stage 2.^17^

The association of GER symptoms with sleep quality highlights the importance of managing GERD to minimize risk of long-term complications. Currently, the most common medical treatment for GERD is acid suppression with administration of a PPI. Randomized placebo-controlled clinical trials have shown that PPI administration results in less nocturnal heartburn and GERD-related sleep disturbances.^27,28^ However, emerging evidence suggests that nonacid reflux may also play a significant role in sleep quality. In a study that included 1415 patients with nonerosive reflux disease, 25% of PPI users whose heartburn improved after the medical therapy still had regurgitation, which contributed to sleep disturbance.^29^ After surgical intervention for GERD, improvement in overall sleep quality measured by the Pittsburgh Sleep Quality Index was observed for 3 months postoperatively.^30^ However, there was no correlation between the improvement in reflux events detected through 24-hour pH monitoring and sleep quality.

### Strengths and Limitations

Our study has several strengths. First, we prospectively collected data on GER symptoms among individuals without a history of sleep disturbances, minimizing potential recall bias and the potential for the direction of the association being reversed. We also had the ability to adjust for known and putative risk factors for poor sleep quality, such as BMI, physical inactivity, diet, and other comorbidities. We also considered whether use of acid-suppressive therapy modified the association between GER symptoms and poor sleep quality.

Study limitations include an inability to distinguish between daytime and nocturnal GER symptoms and a lack of data on objective testing for physiologic reflux to define GERD. However, our assessment of GER symptoms was more clinically relevant as management of GERD primarily relied on self-reported symptoms. A previous study showed considerable discrepancies between the findings from objective testing and patients’ symptoms with regard to sleep problems, in that acid reflux detected by testing was poorly correlated with sleep quality.^30^ Also, due to the nature of the observational study, there may have been residual confounding from unmeasured factors, including comorbidities and psychiatric factors. In addition, we followed up with participants with questionnaires. We also restricted the analysis to participants who returned the 2017 questionnaire and provided information on all domains of sleep quality, and this could have limited the generalizability of the findings.

## Conclusions

Our data showed that GER symptoms may be associated with risk of poor sleep quality among women. Because poor sleep quality has been associated with incidence of chronic disease and mortality,^8,9^ these results further highlight the importance of promptly assessing and effectively treating patients with GERD.

## Notes

Supplement 1. **eMethods**. **eFigure.** Flow Diagram **eTable 1.** Modified PSQI According to the Frequency of Reflux Symptoms **eTable 2.** Modified PSQI According to the Duration of Having GER Symptoms Once or More a Week **eTable 3.** Risk of Poor Sleep According to the Frequency of Reflux Symptoms Within Selected Subgroups **eTable 4.** Risk of Poor Sleep According to the Duration of Having GER Symptoms Within Once or More a Week Within Selected Subgroups

Supplement 2.
Data Sharing Statement
