# Supplementary material for: Assessment of Gastroesophageal Reflux Symptoms and Sleep Quality Among Women in the Nurses’ Health Study II
Source: JAMA Netw Open. 2023 Jul 19;6(7):e2324240. doi: 10.1001/jamanetworkopen.2023.24240 (PMC10357337; doi:10.1001/jamanetworkopen.2023.24240)
Supplement: Supplement 1. — eMethods. eFigure. Flow Diagram eTable 1. Modified PSQI According to the Frequency of Reflux Symptoms eTable 2. Modified PSQI According to the Duration of Having GER Symptoms Once or More a Week eTable 3. Risk of Poor Sleep According to the Frequency of Reflux Symptoms Within Selected Subgroups eTable 4. Risk of Poor Sleep According to the Duration of Having GER Symptoms Within Once or More a Week Within Selected Subgroups [file jamanetwopen-e2324240-s001.pdf]

Supplemental Online Content

Ha J, Mehta RS, Cao Y, Huang T, Staller K, Chan AT. Assessment of gastroesophageal reflux symptoms and sleep quality among women in the Nurses' Health Study II. *JAMA Netw Open*. 2023;6(7):e2324240. doi:10.1001/jamanetworkopen.2023.24240

eMethods.
eFigure. Flow Diagram
eTable 1. Modified PSQI According to the Frequency of Reflux Symptoms Results of Quality Assessment per Study
eTable 2. Modified PSQI According to the Duration of Having GER Symptoms Once or More a Week
eTable 3. Risk of Poor Sleep According to the Frequency of Reflux Symptoms Within Selected Subgroups
eTable 4. Risk of Poor Sleep According to the Duration of Having GER Symptoms Within Once or More a Week Within Selected Subgroups

This supplemental material has been provided by the authors to give readers additional information about their work.

© 2023 Ha J et al. *JAMA Network Open*.

eMethods

Adapted Pittsburgh Sleep Quality Index

We used five domains to calculate the sleep score in accordance with the Pittsburgh Sleep Quality Index based on the questionnaire in 2017, which asked about the sleep quality in the past 4 weeks.[1] Each domain was scored from 0 (better quality) to 3 (worse quality), as described below, and the scores of the five domains were summed up. Poor sleep quality was defined as having a total score higher than 7. In addition to evaluating the risk of poor sleep quality, we investigated the risk of having sleep problems with the individual components with binary outcomes.

Component 1: Difficulty in falling asleep (Sleep latency). Participants answered a question, "Did you have trouble falling asleep", with options of "no" (0 points), "less than once/week" (1 point), "1-2 times per week" (2 points), "3-4 times per week" (3 points), or "5+ times per week" (3 points). Participants were considered as having difficulty falling asleep if they answered "3-4 times per week" or "5+ times per week" for analyses as a binary outcome.
Component 2: Daytime sleepiness. Participants answered a question, "On average, to what extent is your daily functioning (e.g., fatigue, mood, ability to work, concentration, memory) affected by not being well-rested", with options of "not at all" (0 points), "a little" (1 point), "a moderate amount" (2 points), "quite a bit" (2 points), or "very much" (3 points). When evaluating the risk of excessive daytime sleepiness, participants who answered "a moderate amount", "quite a bit", or "very much" were considered to have excessive daytime sleepiness.
Component 3: Restlessness of sleep (Subjective sleep quality). To a question, "Overall, was your typical sleep during the past 4 weeks:", participants answered, "very sound or restful" (0 points), "sound or restful" (1 point), "average quality" (1 point), "restless" (2 points), or "very restless" (3 points). Participants were considered to have restlessness of sleep if they answered "restless" or "very restless".
Component 4: Sleep disturbance. Three questions were asked for sleep disturbance: "Did you wake up several times at night?", "Did you wake up earlier than you planned to?", "Did you have trouble getting back to sleep after you woke up too early?". Points were given for each question according to participants' answers: "no" (0 points), "less than once/week" (1 point), "1-2 times per week" (2 points), "3-4 times per week" (3 points), "5+ times per week" (3 points) and then summed up. An overall score was assigned as 0 for the sum of 0, 1 for the sum of 1 to 3, 2 for the sum of 4 to 6, and 3 for the sum of 7 to 9. To evaluate sleep disturbance as a binary outcome, participants with answering "3-4 times per week" or "5+ times per week" for two or three questions were considered to have sleep disturbance.
Component 5: Sleep duration. Participants were asked, "On average, over a 24-hour period, do you sleep:", and chose answers among "<5 hours" (3 points), "5 hours" (2 points), "6 hours" (1 point), "7 hours" (0 points), "8 hours" (0 points), "9 hours" (0 points), "10+ hours" (0 points). Participants with average sleep of fewer than 7 hours per day were considered to have short sleep duration.

© 2023 Ha J et al. *JAMA Network Open*.

Assessment of covariates

Age, weight, and height were obtained on the questionnaires. Body mass index (BMI) was calculated as weight in kilograms divided by the square of height in meters. Information on smoking history (current, past, or never smoker) and pack-years, alcohol consumption in grams per day, menopausal hormone therapy use (premenopausal status, current user, past user, or never user), and the use of diuretics (thiazide or furosemide) were also updated every two years. Physical activity was derived through questions about different types of exercise in metabolic equivalent task (MET) hours per week. The history of being diagnosed with cancer, congestive heart failure, diabetes, asthma, hyperthyroidism, hypothyroidism, and depression was asked. The presence of urinary incontinence during the last 12 months and the presence of hot flashes or night sweats during the past 4 weeks were asked. Also, questions based on Patient Health Questionnaire 4 (PHQ-4) were asked to evaluate whether participants had time lasting 2 weeks or longer 1) when most of the day they felt sad, empty, or depressed, 2) when most of the day they were very discouraged about how things were going in their life, 3) when they lost interest in most things they usually enjoy like work, hobbies, and personal relationships, 4) when most days they felt much more anxious and worried that other people with the same problems as them. Self-reported depression/ anxiety was evaluated on a scale of 0 to 4 depending on the questions the participant answered "Yes". Average beverage intakes were evaluated using previously validated semiquantitative food frequency questionnaires.[2] Caffeinated (regular coffee, caffeinated tea, low-calorie carbonated beverages with caffeine) and decaffeinated beverages (decaffeinated coffee, decaffeinated tea, low-calorie carbonated beverages without caffeine) intakes were calculated by adding up individual items. Alternative Healthy Eating Index (AHEI-2010) was used to measure diet quality. The cumulative average of BMI, alcohol consumption, beverage intake, and physical activity from 2005 to 2013 was calculated and used for adjustments. Multivariable models were adjusted for age (continuous in years), BMI (continuous in kg/m^2), menopausal status and menopausal hormone use (premenopausal; never user; past user; current user), smoking status (never smoker; past smoker; current smoker), smoking amount (continuous in pack-years), race (white; non-white), presence of cancer (yes; no), congestive heart failure (yes; no), diabetes (yes; no), asthma (yes; no), hyperthyroidism (yes; no), hypothyroidism (yes; no), depression (yes; no), the number of depression/ anxiety symptoms (ordinal), urinary incontinence (yes; no), and hot flushing (yes; no), alcohol consumption (continuous in g/day), intake of caffeinated beverage and decaffeinated beverage (continuous in servings/day), physical activity (continuous in MET/week), proton pump inhibitor (PPI)/ histamine H_2 receptor antagonist (H2RA) use (yes; no), diuretics use (yes; no), the number of months working night shifts (≥1 month; <1 month in the past 2 years), and Alternative Healthy Eating Index (continuous).

References for eMethods

1. Buysse DJ, Reynolds III CF, Monk TH, Berman SR, Kupfer DJ. The Pittsburgh Sleep Quality Index: a new instrument for psychiatric practice and research. *Psychiatry research.* 1989;28(2):193-213.
2. Curhan GC, Willett WC, Knight EL, et al. Dietary factors and the risk of incident kidney stones in younger women: Nurses' Health Study II. *Arch Intern Med.* 2004;164:885-91.

© 2023 Ha J et al. *JAMA Network Open*.

eFigure. Flow Diagram

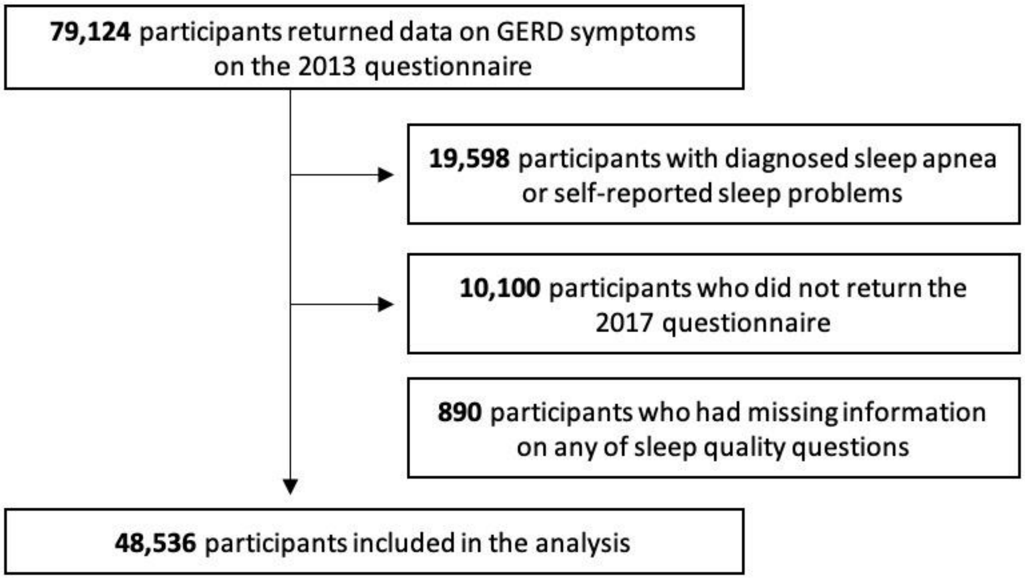

© 2023 Ha J et al. *JAMA Network Open*.

eTable 1. Modified PSQI According to the Frequency of Reflux Symptoms

Score, mean (SD)	Frequency of GER symptoms				P-trend [a]
	**<1/month** (n=28991)	**1-3/month** (n=3436)	**1/week** (n=8383)	**≥2/week** (n=7726)	
Overall score	4.74 (2.46)	5.12 (2.43)	5.36 (2.53)	5.58 (2.66)	<.001
Difficulty in falling asleep	0.93 (1.03)	1.06 (1.05)	1.13 (1.09)	1.18 (1.13)	<.001
Daytime sleepiness	0.70 (0.69)	0.79 (0.69)	0.85 (0.71)	0.90 (0.73)	<.001
Restlessness of sleep	1.04 (0.52)	1.09 (0.50)	1.12 (0.52)	1.16 (0.56)	<.001
Sleep disturbance	1.77 (0.95)	1.87 (0.93)	1.92 (0.92)	1.96 (0.93)	<.001
Sleep duration	0.31 (0.58)	0.31 (0.59)	0.34 (0.63)	0.38 (0.67)	<.001

Abbreviations: BMI, body mass index; GER, gastroesophageal reflux; H2RA, histamine H_2 receptor antagonist; MHT, menopausal hormone therapy; PPI, proton pump inhibitor; PSQI, Pittsburgh Sleep Quality Index; SD, standard deviation

[a] P-trend controlling for age (continuous), BMI (continuous), menopausal status/menopausal hormone use (premenopausal, MHT never user, MHT current user, MHT past user), smoking (never, past, current smoker), race (white, others), presence of cancer (yes, no), congestive heart failure (yes, no), diabetes (yes, no), asthma (yes, no), hyperthyroidism (yes, no), hypothyroidism (yes, no), depression (yes, no), self-reported depression and anxiety symptoms (ordinal; 0 to 4), urinary incontinence (yes, no), and hot flushing (yes, no), alcohol consumption (continuous), intake of caffeinated beverage (continuous) and decaffeinated beverage (continuous), physical activity (continuous), diuretics use (yes, no), H2RA/PPI use (yes, no), the number of months working night shifts (≥1 month, <1 month), and Alternative Healthy Eating Index (continuous).

© 2023 Ha J et al. *JAMA Network Open*.

eTable 2. Modified PSQI According to the Duration of Having GER Symptoms Once or More a Week

Score, mean (SD)	Duration of having GER symptoms once or more a week				P-trend [a]
	**No GERD** (n=28283)	**<4 years** (n=2699)	**4-7 years** (n=3325)	**≥8 years** (n=14229)	
Overall score	4.74 (2.45)	5.22 (2.52)	5.42 (2.57)	5.40 (2.59)	<.001
Difficulty in falling asleep	0.93 (1.03)	1.07 (1.09)	1.13 (1.11)	1.14 (1.11)	<.001
Daytime sleepiness	0.70 (0.69)	0.83 (0.71)	0.86 (0.72)	0.86 (0.72)	<.001
Restlessness of sleep	1.04 (0.52)	1.11 (0.54)	1.13 (0.54)	1.12 (0.53)	<.001
Sleep disturbance	1.77 (0.95)	1.89 (0.94)	1.94 (0.93)	1.91 (0.93)	<.001
Sleep duration	0.31 (0.58)	0.32 (0.61)	0.36 (0.64)	0.36 (0.65)	<.001

Abbreviations: BMI, body mass index; GER, gastroesophageal reflux; H2RA, histamine H_2 receptor antagonist; MHT, menopausal hormone therapy; PPI, proton pump inhibitor; PSQI, Pittsburgh Sleep Quality Index; SD, standard deviation

[a] P-trend controlling for age (continuous), BMI (continuous), menopausal status/menopausal hormone use (premenopausal, MHT never user, MHT current user, MHT past user), smoking (never, past, current smoker), race (white, others), presence of cancer (yes, no), congestive heart failure (yes, no), diabetes (yes, no), asthma (yes, no), hyperthyroidism (yes, no), hypothyroidism (yes, no), depression (yes, no), self-reported depression and anxiety symptoms (ordinal; 0 to 4), urinary incontinence (yes, no), and hot flushing (yes, no), alcohol consumption (continuous), intake of caffeinated beverage (continuous) and decaffeinated beverage (continuous), physical activity (continuous), diuretics use (yes, no), H2RA/PPI use (yes, no), the number of months working night shifts (≥1 month, <1 month), and Alternative Healthy Eating Index (continuous)

© 2023 Ha J et al. *JAMA Network Open*.

eTable 3. Risk of Poor Sleep According to the Frequency of Reflux Symptoms Within Selected Subgroups

Subgroups	Frequency of GER symptoms				P for interaction
	**<1/month**	**1-3/month**	**1/week**	**≥2/week**	
**Age**					.78
**<60 years** (n=26312)	16121	1930	4366	3895	
Multivariable RR (95% CI) [a]	1 [ref]	1.14 (1.03-1.27)	1.21 (1.13-1.31)	1.51 (1.40-1.62)	
**≥60 years** (n=22224)	12870	1506	4017	3831	
Multivariable RR (95% CI) [a]	1 [ref]	1.17 (1.03-1.33)	1.44 (1.33-1.57)	1.56 (1.44-1.70)	
**BMI**					.19
**<25 kg/m^2** (n=22054)	15143	1449	2940	2522	
Multivariable RR (95% CI) [a]	1 [ref]	1.13 (1.00-1.28)	1.36 (1.25-1.48)	1.54 (1.41-1.69)	
**≥25 kg/m^2** (n=26482)	13848	1987	5443	5204	
Multivariable RR (95% CI) [a]	1 [ref]	1.16 (1.05-1.29)	1.27 (1.19-1.36)	1.51 (1.41-1.62)	
**Smoking status**					.40
**Never** (n=32247)	19790	2220	5324	4913	
Multivariable RR (95% CI) [a]	1 [ref]	1.14 (1.03-1.26)	1.32 (1.23-1.41)	1.56 (1.46-1.67)	
**Current/past** (n=16289)	9201	1216	3059	2813	
Multivariable RR (95% CI) [a]	1 [ref]	1.17 (1.03-1.33)	1.29 (1.17-1.41)	1.48 (1.35-1.62)	
**Physical activity**					.45
**<20 MET/wk** (n=24331)	13207	1850	4766	4508	
Multivariable RR (95% CI) [a]	1 [ref]	1.17 (1.05-1.30)	1.24 (1.15-1.30)	1.52 (1.41-1.63)	
**≥20 MET/wk** (n=24205)	15784	1586	3617	3218	
Multivariable RR (95% CI) [a]	1 [ref]	1.11 (0.98-1.26)	1.39 (1.28-1.50)	1.52 (1.40-1.66)	

Abbreviations: BMI, body mass index; GER, gastroesophageal reflux; H2RA, histamine H_2 receptor antagonist; MHT, menopausal hormone therapy; PPI, proton pump inhibitor; RR, relative risk

[a] Multivariable model adjusted for age (continuous), BMI (continuous), menopausal status/menopausal hormone use (premenopausal, MHT never user, MHT current user, MHT past user), smoking (never, past, current smoker), race (white, others), presence of cancer (yes, no), congestive heart failure (yes, no), diabetes (yes, no), asthma (yes, no), hyperthyroidism (yes, no), hypothyroidism (yes, no), depression (yes, no), self-reported depression and anxiety symptoms (ordinal; 0 to 4), urinary incontinence (yes, no), and hot flushing (yes, no), alcohol consumption (continuous), intake of caffeinated beverage (continuous) and decaffeinated beverage (continuous), physical activity (continuous), diuretics use (yes, no), H2RA/PPI use (yes, no), the number of months working night shifts (≥1 month, <1 month), and Alternative Healthy Eating Index (continuous)

© 2023 Ha J et al. *JAMA Network Open*.

eTable 4. Risk of Poor Sleep According to the Duration of Having GER Symptoms Within Once or More a Week Within Selected Subgroups

Subgroups	Duration of having GER symptoms once or more per week				P for interaction
	**None**	**<4 years**	**4-7 years**	**≥8 years**	
**Age**					.02
**<60 years** (n=26312)	15898	1445	1761	7208	
Multivariable RR (95% CI) [a]	1 [ref]	1.23 (1.10-1.38)	1.32 (1.20-1.46)	1.32 (1.20-1.46)	
**≥60 years** (n=22224)	12385	1254	1564	7021	
Multivariable RR (95% CI) [a]	1 [ref]	1.22 (1.06-1.40)	1.47 (1.32-1.65)	1.47 (1.36-1.58)	
**BMI**					.33
**<25 kg/m^2** (n=22054)	14979	1064	1204	4807	
Multivariable RR (95% CI) [a]	1 [ref]	1.25 (1.09-1.43)	1.31 (1.16-1.49)	1.38 (1.28-1.48)	
**≥25 kg/m^2** (n=26482)	13304	1635	2121	9422	
Multivariable RR (95% CI) [a]	1 [ref]	1.20 (1.07-1.34)	1.41 (1.29-1.55)	1.35 (1.27-1.44)	
**Smoking status**					.69
**Never** (n=32247)	19406	1775	2123	8943	
Multivariable RR (95% CI) [a]	1 [ref]	1.26 (1.14-1.40)	1.37 (1.25-1.50)	1.38 (1.30-1.47)	
**Current/past** (n=16289)	8877	924	1202	5286	
Multivariable RR (95% CI) [a]	1 [ref]	1.15 (0.99-1.34)	1.40 (1.24-1.59)	1.33 (1.23-1.44)	
**Physical activity**					.37
**<20 MET/wk** (n=24331)	12820	1505	1858	8148	
Multivariable RR (95% CI) [a]	1 [ref]	1.21 (1.08-1.36)	1.44 (1.30-1.58)	1.34 (1.26-1.43)	
**≥20 MET/wk** (n=24205)	15463	1194	1467	6081	
Multivariable RR (95% CI) [a]	1 [ref]	1.22 (1.07-1.39)	1.29 (1.15-1.45)	1.38 (1.28-1.48)	

Abbreviations: BMI, body mass index; GER, gastroesophageal reflux; H2RA, histamine H_2 receptor antagonist; MHT, menopausal hormone therapy; PPI, proton pump inhibitor; RR, relative risk

[a] Multivariable model adjusted for age (continuous), BMI (continuous), menopausal status/menopausal hormone use (premenopausal, MHT never user, MHT current user, MHT past user), smoking (never, past, current smoker), race (white, others), presence of cancer (yes, no), congestive heart failure (yes, no), diabetes (yes, no), asthma (yes, no), hyperthyroidism (yes, no), hypothyroidism (yes, no), depression (yes, no), self-reported depression and anxiety symptoms (ordinal; 0 to 4), urinary incontinence (yes, no), and hot flushing (yes, no), alcohol consumption (continuous), intake of caffeinated beverage (continuous) and decaffeinated beverage (continuous), physical activity (continuous), diuretics use (yes, no), H2RA/PPI use (yes, no), the number of months working night shifts (≥1 month, <1 month), and Alternative Healthy Eating Index (continuous)

© 2023 Ha J et al. *JAMA Network Open*.
