# Supplementary material for: Assessment of Gastroesophageal Reflux Symptoms and Sleep Quality Among Women in the Nurses’ Health Study II
Source: JAMA Netw Open. 2023 Jul 19;6(7):e2324240. doi: 10.1001/jamanetworkopen.2023.24240 (PMC10357337; doi:10.1001/jamanetworkopen.2023.24240)
Supplement: Supplement 2. — Data Sharing Statement [file jamanetwopen-e2324240-s002.pdf]

Data Sharing Statement

Ha. Assessment of Gastroesophageal Reflux Symptoms and Sleep Quality Among Women in the Nurses' Health Study II. *JAMA Netw Open*. Published July 19, 2023. doi:10.1001/jamanetworkopen.2023.24240

Data
Data available: Yes
Data types: Deidentified participant data
How to access data: Information including the procedures to obtain and access data from the Nurses' Health Studies is described at https://www.nurseshealthstudy.org/researchers (contact email: nhsaccess@channing.harvard.edu)
When available: With publication

Supporting Documents
Document types: None

Additional Information
Who can access the data: Researchers whose proposed use of the data has been approved
Types of analyses: For a specified purpose
Mechanisms of data availability: with investigator support, after approval of a proposal, with a signed data access agreement
